# Beyond Biology: Uncovering Structural and Sociocultural Predictors of Breast Cancer Incidence Worldwide

**DOI:** 10.3390/curroncol32100553

**Published:** 2025-10-02

**Authors:** Janet Diaz-Martinez, Gustavo A. Hernández-Fuentes, Josuel Delgado-Enciso, Mario A. Alcalá-Pérez, Isaac Jiménez-Calvo, Carmen A. Sánchez-Ramírez, Fabian Rojas-Larios, Alejandrina Rodriguez-Hernandez, Mario Ramírez-Flores, José Guzmán-Esquivel, Karmina Sánchez-Meza, Ana C. Espíritu-Mojarro, Osval A. Montesinos-López, Iván Delgado-Enciso

**Affiliations:** 1 Department of Dietetics and Nutrition, Research Center in Minority Institutions, Florida International University (FIU-RCMI), Miami, FL 33199, USA; jdimarti@fiu.edu; 2 Department of Molecular Medicine, School of Medicine, University of Colima, Colima 28040, Mexico; carmen_sanchez@ucol.mx (C.A.S.-R.); frojas@ucol.mx (F.R.-L.); arodrig@ucol.mx (A.R.-H.); mario_ramirez@ucol.mx (M.R.-F.); ksmeza@ucol.mx (K.S.-M.); 3 State Cancerology Institute of Colima, Health Services of the Mexican Social Security Institute for Welfare (IMSS-BIENESTAR), Colima 28085, Mexico; 21460570@colima.tecnm.mx; 4 Faculty of Chemical Sciences, University of Colima, Coquimatlan 28400, Mexico; 5 Foundation for Ethics Education and Cancer Research of the IEC of Colima AC, Colima 28085, Mexico; derecho@ucol.mx; 6 Molecular Medicine Laboratory, Unidad de Medicina Humana y Ciencias de la Salud, Universidad Autónoma de Zacatecas, Zacatecas 98160, Mexico; marioalcalaperez@uaz.edu.mx; 7 Clinical Epidemiology Research Unit, Mexican Institute of Social Security, Villa de Alvarez 28984, Mexico; jose.esquivel@imss.gob.mx; 8 Department of Pediatrics, Mexican Institute of Social Security (IMSS), General Hospital of Zone No. 1, Villa de Alvarez 28984, Mexico; aespiritu3@ucol.mx; 9 Faculty of Telematics, University of Colima, Colima 28040, Mexico; oamontes1@ucol.mx; 10 Robert Stempel College of Public Health and Social Work, Florida International University, Miami, FL 33199, USA

**Keywords:** sociodemographic factors, breast cancer, sociocultural factors, environmental factors, level of unhealthiness, healthcare expenditure, educational level

## Abstract

**Simple Summary:**

Breast cancer is one of the most common and deadly cancers worldwide, and its occurrence varies greatly between countries. While biological factors are well-known, this study aimed to explore how social, economic, and environmental conditions may also influence breast cancer rates. By analyzing global data from 183 countries, researchers found that factors like low breastfeeding rates, cocaine use, poor sanitation, high out-of-pocket health costs, and diets rich in processed meats were linked to higher breast cancer incidence. Surprisingly, the lack of basic hygiene facilities was also strongly associated, but in unexpected ways. These findings suggest that improving public health systems, encouraging healthier lifestyles, and addressing broader social conditions could help lower breast cancer rates worldwide. This research offers new perspectives that may guide future studies, shape health policies, and support more effective prevention strategies.

**Abstract:**

Breast cancer remains a leading cause of global cancer burden, with marked differences in incidence across countries. While biological risk factors are well established, understanding the broader structural and sociocultural influences has been less comprehensive. In this study, we analyzed harmonized data from 183 countries (2017–2023), encompassing 33 variables and 7 subvariables related to demographics, nutrition, environment, health, and healthcare access, drawn from open-access international databases. Spearman correlation analysis identified strong positive associations between breast cancer incidence and discontinued breastfeeding, high LDL cholesterol, out-of-pocket healthcare expenditure, and educational attainment. Conversely, poor sanitation, lack of handwashing facilities, unsafe water, and certain nutritional deficiencies exhibited robust negative correlations, likely reflecting under detection and reporting limitations in lower-resource settings rather than true protective effects. These findings were further explored using multiple linear regression, which explained approximately 73% of the variance in global breast cancer incidence. The final model highlighted discontinued breastfeeding, prevalence of cocaine use, unsafe sanitation, high out-of-pocket healthcare expenditure, limited handwashing access, and high processed meat consumption as the most influential independent predictors. Receiver operating characteristic (ROC) analysis confirmed strong predictive value for discontinued breastfeeding and out-of-pocket expenditure, with sanitation and hygiene variables showing paradoxical inverse associations. Our results emphasize that breast cancer risk is shaped not only by individual behaviors and genetics, but also by larger-scale structural, socioeconomic, and environmental factors. These patterns suggest that targeted interventions addressing both lifestyle behaviors and systemic inequities—such as promoting breastfeeding, reducing financial barriers to healthcare, and strengthening public health infrastructure—could meaningfully reduce the global burden of breast cancer. In conclusion, this study underscores the importance of multisectoral, equity-focused prevention strategies. It also highlights the value of country-level ecological analyses in uncovering upstream determinants of cancer incidence and calls for further research to disentangle individual and contextual effects in cancer epidemiology.

## 1. Introduction

Breast cancer is the focal point of our research, not only due to its significant clinical burden but also because of its far-reaching social implications [1]. Understanding this complexity is crucial for developing effective strategies for cancer prevention, early detection, and treatment, underscoring the ongoing global efforts to combat this pervasive health threat [2]. This public health issue transcends geographical boundaries, affecting women from diverse backgrounds globally. According to the 2020 GLOBOCAN report, breast cancer is the most prevalent cancer among women in 159 of the 183 countries analyzed and the leading cause of cancer-related mortality in women in 110 of these countries [3]. Furthermore, the incidence of breast cancer has been documented to progressively increase, with variations observed across different geographical regions [3,4]. These trends underscore the need for region-specific strategies to address the growing burden of breast cancer worldwide.

A key aspect in understanding the global patterns of breast cancer is identifying the sociodemographic factors that influence its incidence. These include population-level characteristics such as age, sex, education, income, occupation, and healthcare access—factors that can significantly shape health outcomes, including cancer risk. Understanding how these elements contribute to differences in breast cancer rates is essential to explain regional disparities and guide more effective prevention and public health strategies [5,6].

Differences in cancer incidence across the globe are closely linked to the specific characteristics of populations residing in different geographical regions. Cancer has long been recognized as a multifactorial disease, with its development influenced by a range of modifiable and non-modifiable factors [7]. Landmark studies by Doll and Peto in 1981, and later by Clapp and colleagues in 2007, have demonstrated that a combination of environmental, occupational, lifestyle, dietary, and other factors is implicated in the causation of approximately 80% of cancers [8,9]. These findings highlight the importance of examining broader contextual influences alongside biological mechanisms [10,11].

The objective of this study is to examine how diverse sociodemographic, environmental, and lifestyle contexts interact to shape global patterns of breast cancer incidence. Using data from 183 countries and adopting a macro-level perspective, this work seeks to identify overlooked influences and provide new insights to guide more context-specific prevention and public health strategies. The dataset integrates a wide range of variables—including environmental, geographic, epidemiological, cultural, and social indicators—that help reveal multifaceted patterns of breast cancer incidence at the population level [10,11]. Moreover, this study explores how previously identified contextual variables may interact with one another, aiming to advance a comprehensive understanding of how sociodemographic, environmental, and lifestyle factors jointly shape global patterns of disease.

## 2. Materials and Methods

### 2.1. Data Collection and Selection

An extensive review was conducted using international and publicly accessible databases [5,6,7,8,9,10,11,12,13,14,15,16,17,18]. covering the period from 2019 to 2023. A total of 33 primary nutritional and socioeconomic variables, along with 7 relevant subvariables, were systematically collected across 183 countries to capture a broad spectrum of modifiable and non-modifiable factors potentially contributing to breast cancer risk, resulting in 40 variables analyzed in total (Table 1).

Modifiable variables include lifestyle habits (e.g., alcohol consumption, tobacco use, physical inactivity), nutritional factors (e.g., intake of fruits, vegetables, sugar, fats, dairy, sodium), health indicators (e.g., BMI, blood pressure, fasting glucose), environmental and hygiene-related exposures (e.g., access to clean water, sanitation, and handwashing facilities), and access to healthcare services (e.g., out-of-pocket healthcare spending).

All variables included in this study were selected based on their documented relevance to breast cancer incidence and were obtained from reputable international institutions, including the World Health Organization (WHO), the Institute for Health Metrics and Evaluation (IHME), the Food and Agriculture Organization (FAO), the United Nations Development Programme (UNDP), and the United Nations Office on Drugs and Crime (UNODC). Where applicable, data were standardized to enable cross-country comparisons using consistent methodological criteria [12]. Table 1 provides a categorized overview of all variables, along with their definitions and corresponding data sources.

### 2.2. Inclusion and Exclusion Criteria

A systematic search was conducted in freely accessible online databases (sources included the Global Cancer Observatory (GCO, IARC/WHO) for cancer incidence, the United Nations Development Programme (UNDP) for sociodemographic indicators, the World Health Organization (WHO) and World Bank for healthcare expenditures, the Institute for Health Metrics and Evaluation (IHME) and Global Burden of Disease Study (GBD) for health-related and nutritional risk factors, the United Nations Office on Drugs and Crime (UNODC) for substance use, the Food and Agriculture Organization of the United Nations (FAO) for dietary supply data, and Our World in Data (OWID) as a secondary processor of UN and WHO datasets. Variables that did not have global coverage or were not disaggregated by sex were excluded) to collect quantifiable data on the study variables. Initially, Boolean search terms such as “AND,” “OR,” “breast cancer,” “sociodemographic factors,” “risk factors,” “nutritional factor,” “healthcare system,” “per capita,” and “food” were employed to identify studies and databases providing global information covering at least 183 countries.

One key inclusion criterion was that the data had to be current, specifically published between 2019 and 2023, to ensure relevance and timeliness. This timeframe was selected because several databases had not been recently updated, partly due to governmental, political, or economic limitations affecting data availability.

Following this broad search strategy, a second targeted search was conducted within those databases meeting the inclusion criteria, ensuring the accessibility and global coverage of the selected variables.

Studies or databases were excluded if they did not provide worldwide data, lacked full accessibility, or contained outdated information outside the specified timeframe.

The primary outcome of this study was to identify and analyze statistical associations between the selected sociodemographic, environmental, health-related, and lifestyle variables and the global incidence of breast cancer. Therefore, only datasets allowing for such correlation analyses with incidence rates were considered eligible for inclusion.

### 2.3. Data Analysis

Quantitative data for each variable were collected from all countries included in the study, resulting in the development of an extensive database that served as the foundation for subsequent analysis [24]. For the dependent variable, age-standardized incidence rates (ASR) of breast cancer were employed. ASR is a measure commonly used in epidemiology and public health to compare disease rates across different populations, accounting for variations in age distribution within those populations. This approach enables a more reliable comparative analysis across diverse groups [25,26].

Statistical analyses were conducted concurrently using SPSS Statistics, version 27.0 (IBM Corp., Armonk, NY, USA, 2021) and SciPy, version 1.7.1 (Python Software Foundation, 2021) to ensure robust results [27]. Initially, the Kolmogorov–Smirnov test was applied to assess data normality, given the large sample size. Upon confirming that the data did not follow a normal distribution, a non-parametric approach was adopted, utilizing Spearman’s correlation to examine the relationship between all sociodemographic factors and Age-standardized incidence rates of breast cancer [27].

#### 2.3.1. Correlation Analysis with FDR Adjustment

Spearman’s rho correlation was employed to assess the associations between sociodemographic, health-related, environmental, and lifestyle variables with age-standardized incidence rates of breast cancer. Given the large number of pairwise comparisons, the Benjamini–Hochberg procedure was subsequently applied to adjust the obtained *p*-values, controlling the False Discovery Rate (FDR). This two-step approach ensured that the correlations identified were statistically significant while reducing the risk of false positives due to multiple testing [28,29].

#### 2.3.2. Incorporation of R and R-Squared (R^2^) Values

A multiple linear regression model was subsequently developed for factors that exhibited a strong and significant correlation with the dependent variable, breast cancer incidence, to achieve the best predictive model. The values of R, R^2^, and the significance of the F-test were calculated and analyzed to quantify the strength and goodness of fit of the various mathematical models. Variables with the highest significance in the beta coefficient analysis and *t*-test significance were selected to construct a more robust model for predicting breast cancer incidence [30].

#### 2.3.3. Mathematical Modeling

After developing a more robust mathematical model, a comparison of means was conducted using the paired Student’s *t*-test to determine if there was a statistically significant difference between these means. Although the *t*-test is typically associated with parametric data, it can be valid for non-parametric scenarios when the sample size is large enough to mitigate violations of normality. This step was crucial to validate the model’s robustness and to ensure its suitability for predicting the dependent variable, breast cancer incidence, based on the independent variables under study [30,31].

#### 2.3.4. Receiver Operating Characteristic (ROC)

Receiver Operating Characteristic (ROC) curves are commonly used to assess the accuracy of diagnostic tests; however, in this study, ROC curves were employed as a final step to evaluate the performance and effectiveness of the resulting multiple linear regression equation. The areas under the ROC curve (AUCs) were calculated for the different variables, along with their 95% confidence intervals, cut-off points, *p*-values, sensitivity, specificity, and predictive values. Predictive capacity was classified based on AUC values as follows: 0.50–0.60 (failed), 0.61–0.70 (worthless), 0.71–0.80 (poor), 0.81–0.90 (good), and >0.90 (excellent) [32,33]. The cut-off point was determined at the point on the curve that maximized sensitivity and specificity [34]. Sensitivity and specificity were classified as follows: high (>80%), moderate (65–80%), and low (<65%) [35,36]. Notably, some variables yielded AUC values below 0.5 but remained statistically significant. For interpretability, two variables originally defined in negative form—Unsafe Sanitation and No Access to Handwashing Facility—were transformed into their positive counterparts (Safe Sanitation and Access to Handwashing Facility) when calculating the ROC curves. This adjustment was performed only for the AUC analysis, ensuring that higher values consistently indicated better conditions, which shifted the AUC values above 0.5 and made the results easier to interpret. All other analyses (e.g., correlations, regressions) were conducted using the original variable definitions [30,32,34,37,38].

### 2.4. Ethical Considerations

This study adheres to ethical guidelines concerning data privacy in the collection, handling, and analysis of the data [35]. As it utilizes publicly available data where no individual patients are identifiable, it does not require approval from an Institutional Review Board, in accordance with national guidelines and those of academic institutions [39,40,41]. However, all authorships have been duly registered (Act on Research Ethics Review of Health Research Projects. Act No. 593, 2011. Available at: Act on Research Ethics Review of Health Research Projects. Act No 593, 2011. 2011. Available at: https://leap.unep.org/countries/dk/national-legislation/act-no-593-relative-ethical-medical-research, accessed on 22 December 2021) [42,43,44,45].

### 2.5. Limitations and Assumptions

Potential limitations identified in this study include the availability of data over the years, as some referenced databases do not always retain previous datasets when updating their information. Additionally, the quality and scope of the selected sociodemographic factors were considered as limiting factors. We acknowledge the assumptions made during the mathematical modeling process and their potential impact on the results.

## 3. Results

A data matrix consisting of 183 countries and 33 variables was analyzed, yielding a total of 6039 data points. The variables were classified into four main categories: epidemiological (1 variable, 3.03%), social and health-related (7 variables, 21.21%), environmental (3 variables, 9.09%), and nutritional (22 variables, 66.67%). The results showed that most of the variables were concentrated on the nutritional and social-health domains. The social and health-related category included variables such as expected years of schooling, out-of-pocket healthcare expenditure, drug use, exposure to unsafe water, inadequate sanitation, lack of access to handwashing facilities, and prevalence of cocaine use. In contrast, the nutritional category encompassed a broader range of variables, including alcohol consumption, body mass index (BMI), high fasting plasma glucose, LDL cholesterol, high blood pressure, low bone mineral density, kidney dysfunction, and various nutritional deficiencies (iron, zinc, vitamin A), as well as dietary patterns (consumption of fruits, vegetables, cereals, sugars, dairy, fats, red and processed meats, sugar-sweetened beverages, omega-3 and polyunsaturated fatty acids), and breastfeeding indicators.

### 3.1. Correlation Analysis

**Figure 1:**
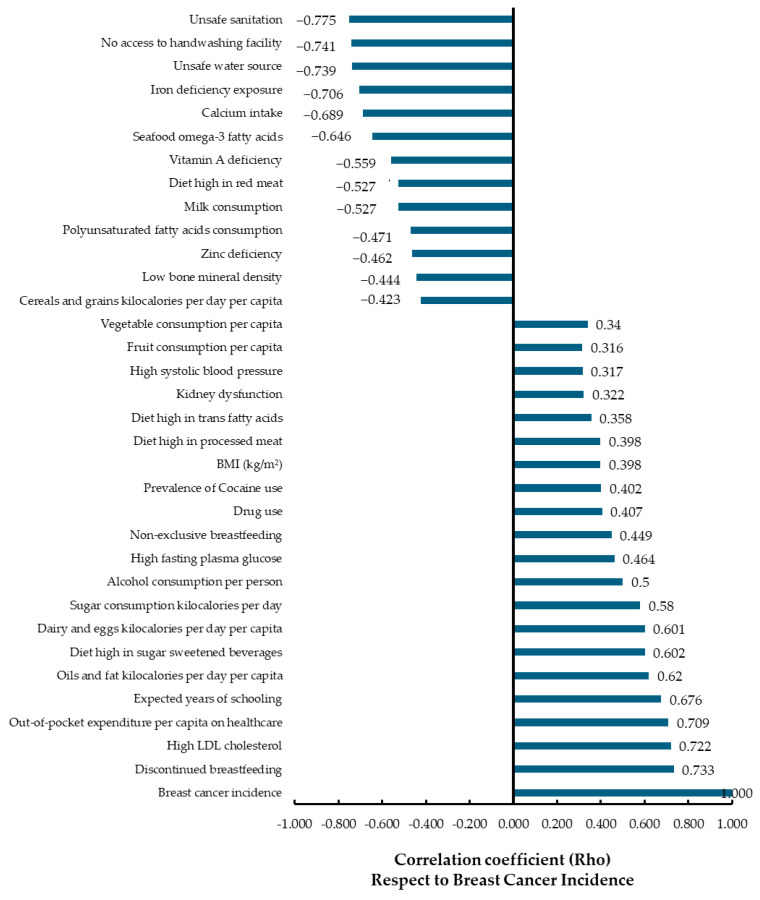
Spearman Correlation Analysis of Environmental and Sociodemographic Factors Associated with Breast Cancer Incidence. Each correlation coefficient reflects the strength and direction of the association. A positive correlation indicates that as one factor increases (discontinued breastfeeding), breast cancer incidence also tends to increase, while a negative correlation suggests the opposite. Significance was determined at a threshold of *p* < 0.05 after False Discovery Rate (FDR) correction.

Figure 1 presents the results of the Spearman correlation analysis, which explored associations between various sociodemographic, environmental, health-related, and nutritional factors and the incidence of breast cancer. An absolute correlation coefficient (ρ) of 0.3 was considered indicative of a weak correlation, while values of ≥ 0.6 were interpreted as strong correlations. A total of 40 variables were included in the correlation analysis to assess their association with breast cancer incidence across the studied population. Out of these, 33 variables demonstrated statistically significant correlations (*p* < 0.05), while 7 variables did not reach statistical significance and were therefore excluded from subsequent multiple regression models. The variables that did not show significant correlation with breast cancer incidence were: passive smoking (r = −0.064, *p* = 0.397), low-fiber diet (r = 0.123, *p* = 0.103), low birth weight and short gestation (r = 0.107, *p* = 0.158), low temperature climate (r = 0.185, *p* = 0.014), occupational exposure to benzene (r = 0.046, *p* = 0.542), high-sodium diet (r = 0.085, *p* = 0.260), and low intake of nuts and seeds (r = 0.038, *p* = 0.619).

Positive correlations with breast cancer incidence were identified in 26 variables, suggesting factors potentially associated with increased risk at the population level. The strongest positive correlations were observed for discontinued breastfeeding (ρ = 0.733), high LDL cholesterol (ρ = 0.722), out-of-pocket healthcare expenditure per capita (ρ = 0.709), and expected years of schooling (ρ = 0.676). Other variables with moderate to weak positive correlations included oils and fats consumption (ρ = 0.620), sugar-sweetened beverage consumption (ρ = 0.602), dairy and eggs consumption (ρ = 0.601), alcohol consumption per person (ρ = 0.500), high fasting plasma glucose (ρ = 0.464), and non-exclusive breastfeeding (ρ = 0.449).

Negative correlations with breast cancer incidence were observed mainly in variables often reflecting socioeconomic or cultural contexts, rather than direct biological protective factors—highlighting the ecological nature of this study. The strongest negative correlations included unsafe sanitation (ρ = −0.775), no access to handwashing facilities (ρ = −0.741), unsafe water source (ρ = −0.739), iron deficiency exposure (ρ = −0.706), and low calcium intake (ρ = −0.689). Other variables with moderate negative correlations were seafood omega-3 fatty acids (ρ = −0.646), vitamin A deficiency (ρ = −0.559), diet high in red meat (ρ = −0.527), milk consumption (ρ = −0.527), and polyunsaturated fatty acid intake (ρ = −0.471).

Additionally, variables such as drug use (ρ = 0.407), cocaine use (ρ = 0.402), BMI (ρ = 0.398), diet high in processed meat (ρ = 0.398), vegetable consumption per capita (ρ = 0.340) and diet high in trans fats (ρ = 0.358) showed weak but positive correlations. Conversely, cereals and grains kilocalories per day (ρ = −0.423) were negatively associated.

It is important to note that all correlations presented in Figure 1 were statistically significant (*p* < 0.05) applying the FDR method to control false discovery rate in our correlation analyses, including both positive and negative associations. The variable “breast cancer incidence” was used as the reference point (ρ = 1.000).

### 3.2. Multiple Regression Analysis

After conducting a correlation analysis, only variables showing a statistically significant relationship (*p* < 0.05) with breast cancer incidence were selected for inclusion in the multiple regression models. A total of six multiple linear regression models were generated, and their performance is summarized in Table 2. Each model includes progressively more predictors, allowing for a stepwise comparison of statistical robustness.

Table 2 summarizes key statistics for each model, including the correlation coefficient (R), coefficient of determination (R^2^), adjusted R^2^, and standard error of the estimate. Model 6 showed the highest adjusted R^2^ (0.721), indicating that approximately 73% of the variance in breast cancer incidence is explained by this combination of predictors.

The variables included in Model 6 were discontinued breastfeeding, prevalence of cocaine use, unsafe sanitation, out-of-pocket healthcare expenditure per capita, lack of access to handwashing facilities, and a diet high in processed meat—all statistically significant at *p* < 0.05.

When examining the individual contribution of each predictor, discontinued breastfeeding was associated with a significant increase in incidence (B = 0.854, 95% CI: 0.384–1.325, *p* < 0.001). Similarly, the prevalence of cocaine use showed a strong positive association (B = 6.934, 95% CI: 4.377–9.491, *p* < 0.001). In contrast, unsafe sanitation was negatively associated with breast cancer incidence (B = −0.337, 95% CI: −0.472 to −0.202, *p* < 0.001). Out-of-pocket healthcare expenditure per capita also contributed positively (B = 0.015, 95% CI: 0.006–0.023, *p* = 0.001), as did lack of access to handwashing facilities (B = 0.129, 95% CI: 0.018–0.241, *p* = 0.023). Finally, a diet high in processed meat was found to be a significant but smaller predictor (B = 0.107, 95% CI: 0.009–0.206, *p* = 0.033).

**Figure 2:**
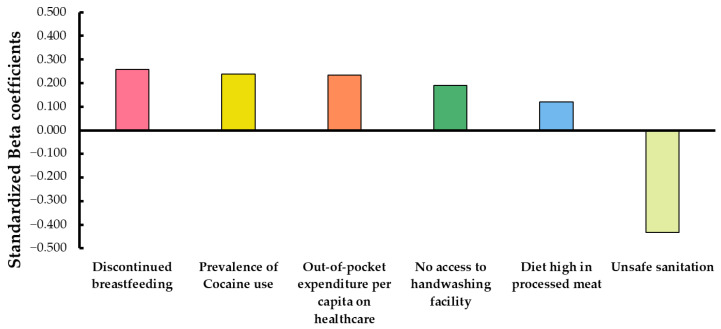
Standardized Beta coefficients from the final multivariate model predicting breast cancer incidence. Bars represent the relative strength and direction of each variable’s association. A “unit increase” means a one-standard-deviation rise in the predictor variable (for example, an increase in average discontinued breastfeeding or Prevalence of Cocaine use), showing how strongly it is linked to changes in breast cancer incidence, either positively or negatively.

To evaluate the relative contribution of each predictor, the standardized Beta coefficients were examined (Figure 2). Among the predictors, unsafe sanitation (β = −0.432) and discontinued breastfeeding (β = 0.257) showed the strongest relative effects on breast cancer incidence.

These findings, graphically summarized in Figure 2, illustrate the differential impact of each variable when controlling for the others, providing a clear picture of their relative importance within the model. Although the standardized Beta coefficients provide insights into the relative importance of each variable in the multivariate context, they do not directly translate into attributable risk at the population level. For such estimates, specific epidemiological models or population attributable fraction (PAF) calculations would be required.

### 3.3. Model Validation

**Figure 3:**
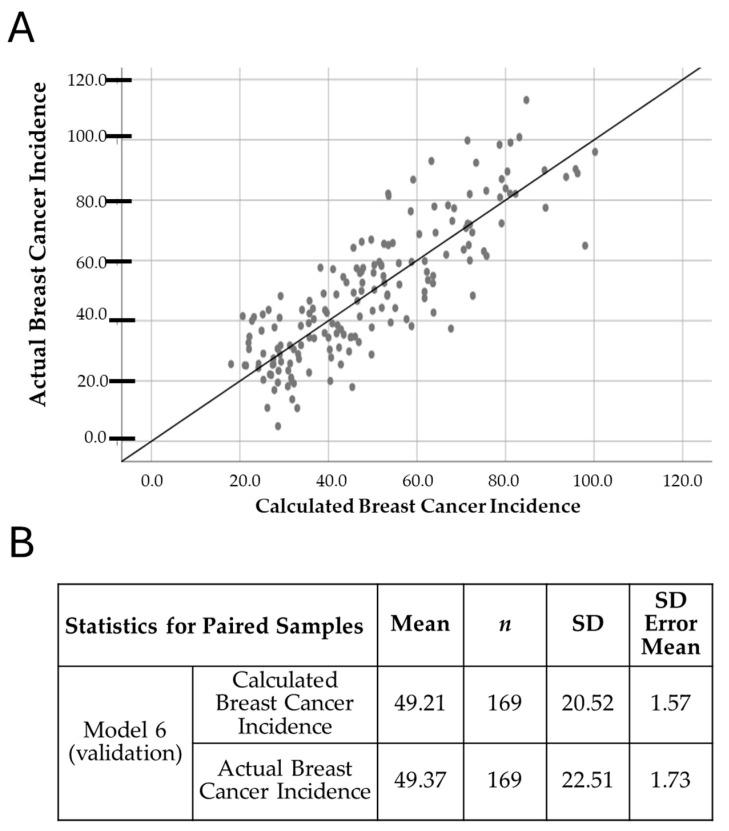
Overview of the breast cancer incidence analysis. It includes a scatter plot obtained after fitting the data with mathematical model 6. (**A**) Scatter plot showing observed data fitted with mathematical model 6, illustrating the agreement between predicted and actual incidence values. (**B**) Results of the paired Student’s *t*-test comparing actual and predicted means. A “unit increase” in this context refers to one step up in the predictor variables, which helps illustrate how each change translates into differences between predicted and observed incidence.

Model 6, identified as the best-performing model from the multiple linear regression analysis, incorporates key sociodemographic and health-related predictors: discontinued breastfeeding, prevalence of cocaine use, unsafe sanitation, out-of-pocket health expenditure per capita, lack of access to handwashing facilities, and a diet high in processed meat. The resulting mathematical model is expressed as follows:

Breast Cancer Incidence = 29.396 + (0.893 × Discontinued Breastfeeding) + (7.273 × Prevalence of Cocaine Use) + (−0.369 × Unsafe Sanitation) + (0.013 × Out-of-pocket Expenditure per Capita on Healthcare) + (0.161 × No Access to Handwashing Facility) + (0.123 × Diet High in Processed Meat). Interpretation of the coefficients is as follows: Discontinued Breastfeeding: Each unit increase leads to an increase of 0.893 in breast cancer incidence. Prevalence of Cocaine Use: Each unit increase results in a rise of 7.273 in breast cancer incidence. Unsafe Sanitation: Each unit increase results in a decrease of 0.369 in breast cancer incidence. Out-of-pocket Expenditure on Healthcare: Each unit increase leads to an increase of 0.013 in breast cancer incidence. No Access to Handwashing Facility: Each unit increase contributes to an increase of 0.161 in breast cancer incidence. Diet High in Processed Meat: Each unit increase results in an increase of 0.123 in breast cancer incidence. Model validation showed a strong agreement between the predicted and actual values of breast cancer incidence, as illustrated in Figure 3A. Furthermore, a comparison of means using Student’s *t*-test (Figure 3B) revealed a negligible absolute difference of only 0.32% between actual and predicted values. This minimal deviation underscores the robustness and predictive accuracy of Model 6, confirming its suitability for estimating the behavior of breast cancer incidence based on the selected variables.

Considering the variables included in the model and their potential impact on breast cancer incidence, we evaluated their discriminatory capacity using the area under the ROC curve (AUC).

**Figure 4:**
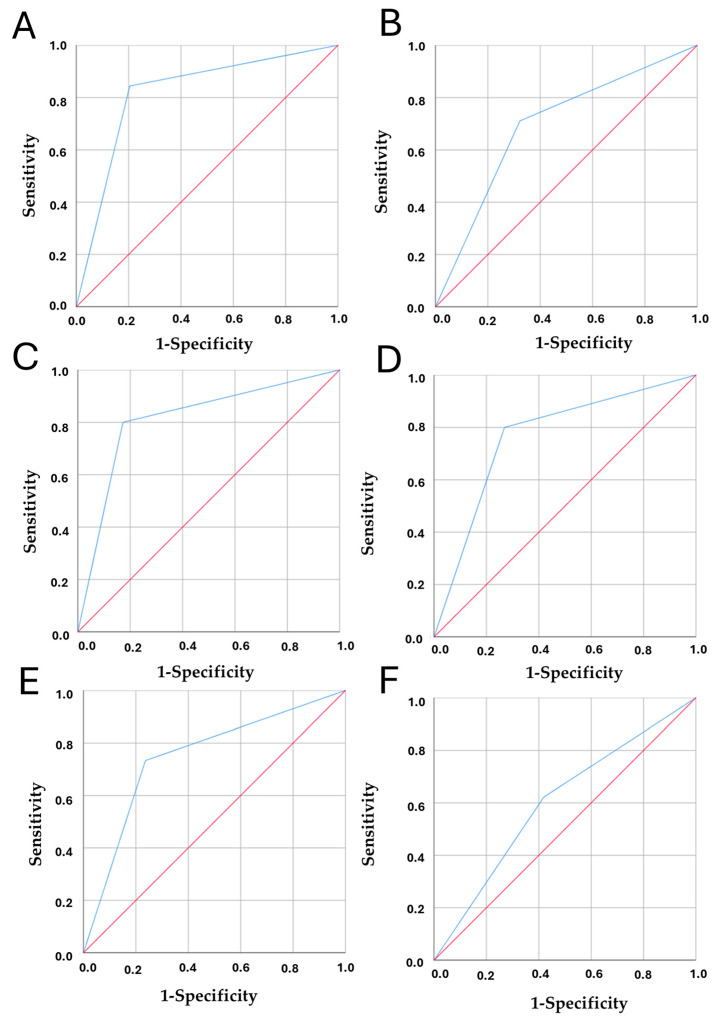
Receiver operating characteristic (ROC) curves showing the discriminatory capacity of the following variables in relation to breast cancer incidence. Notes: The blue line represents the ROC curve for each variable, while the red diagonal line corresponds to the reference line of no discrimination (AUC = 0.5). (**A**) Discontinued breastfeeding, (**B**) Prevalence of cocaine use, (**C**) Safe sanitation, (**D**) Out-of-pocket expenditure per capita on healthcare, (**E**) Access to handwashing facility, and (**F**) Diet high in processed meat. The area under the curve (AUC) is shown for each variable, indicating their ability to differentiate between individuals with and without the outcome. Panels (**C**) (safe sanitation) and (**E**) (access to handwashing facility) represent the counterparts of the original variables (unsafe sanitation and lack of access to handwashing facilities).

“Discontinued of breastfeeding” showed the highest AUC (0.820; 95% CI: 0.756–0.884; *p* < 0.001), indicating strong predictive performance. “Out-of-pocket expenditure per capita on healthcare” also demonstrated good discriminatory power (AUC = 0.766; 95% CI: 0.695–0.837; *p* < 0.001), while “prevalence of cocaine use” showed moderate accuracy (AUC = 0.694; 95% CI: 0.617–0.772; *p* < 0.001). Finally, “diet rich in processed meat” showed limited but statistically significant discriminatory capacity (AUC = 0.601; 95% CI: 0.519–0.684; *p* = 0.042) Figure 4.

Safe Sanitation and Access to Handwashing Facility showed significant discriminatory performance in predicting high breast cancer incidence. Specifically, Safe Sanitation had an AUC of 0.814 (95% CI: 0.7499–0.879; *p* = 0.033), and Access to Handwashing Facility had an AUC of 0.748 (95% CI: 0.676–0.821; *p* = 0.001). Higher values consistently reflect better conditions, indicating that reduced access to sanitation and hygiene is associated with higher breast cancer incidence. These findings likely reflect structural limitations in healthcare access, underreporting, or reduced detection capacity rather than a protective effect, highlighting important social and environmental vulnerabilities.

## 4. Discussion

Understanding global patterns of breast cancer incidence requires more than identifying isolated risk factors; it calls for a comprehensive perspective that considers how biological, social, and structural dimensions interact across diverse contexts. This study diverges from conventional approaches by analyzing breast cancer incidence at the national level, treating each country as an individual epidemiological and sociocultural unit. While acknowledging differences in climatic, demographic, and economic factors [11,46,47,48,49,50], this design also enables the incorporation of anthropological perspectives—examining how breast cancer is shaped by deeper social structures, cultural norms, and historical trajectories. Such a multidimensional view is essential to understand why some countries exhibit higher or lower incidence not only due to exposure to typical risk factors, but also due to how societies live, age, reproduce, eat, and interpret illness [5,47,51].

From a general perspective, clear differences emerge between countries with high and low levels of breast cancer incidence. However, the causal patterns underlying these disparities are not always straightforward, suggesting that multiple, interrelated factors are at play. A comparative analysis reveals a stark contrast in breast cancer incidence rates across countries. The top ten countries with the highest incidence of breast cancer include Belgium (113.2), Luxembourg (99.8), and the Netherlands (100.9), highlighting a significant burden in Western Europe. In stark contrast, the countries with the lowest incidence rates, such as Bhutan (5), Mongolia (11.1), and Gambia (11), suggest a markedly different epidemiological profile. While part of this variation may stem from underdiagnosis and limited healthcare infrastructure in low-income regions, a purely structural explanation is insufficient. In many low-incidence countries, women’s reproductive lives follow different patterns—earlier childbearing, multiple pregnancies, and extended breastfeeding—which not only protect against breast cancer biologically but also reflect embedded cultural ideals of femininity, motherhood, and intergenerational caregiving. In these contexts, breastfeeding is often less a personal health choice and more a socially reinforced expectation, shaped by tradition rather than public health campaigns [48,49,50]. This study does not aim to function as an individualized risk calculator, such as the Gail Model [52], but instead provides a broader, population-based framework for identifying predictors of breast cancer incidence [53,54]. Unlike clinical risk models that focus on individual history and genetics, our approach incorporates sociodemographic, dietary, and structural variables that shape risk at the population level.

In contrast, the model developed in this study explains 73% of the variance in breast cancer incidence across countries by integrating factors such as discontinued breastfeeding, prevalence of cocaine use, high consumption of processed meats and out-of-pocket healthcare expenditure. These variables reflect not only individual behaviors but also broader systemic and cultural dynamics. Thus, although this model is not intended for direct clinical application at the individual level, it offers valuable insight into macro-level determinants of breast cancer, many of which are overlooked in conventional clinical risk models. These findings underscore the importance of including contextual, historical, and structural dimensions—such as urbanization, dietary shifts, and changing maternal roles—as essential considerations in future cancer risk assessment frameworks.

The negative associations with sanitation and handwashing facilities are most likely explained by underdiagnosis and underreporting in resource-limited settings. Certain cultural or lifestyle factors—such as traditional reproductive behaviors, subsistence-based diets, or distinct health ecologies—may also contribute. However, these patterns should not be misinterpreted as protective effects; rather, they represent context-specific dynamics that warrant further study [55]. Populations in these environments often rely on subsistence-based food systems and engage in high levels of physical activity through labor-intensive work. They also tend to consume fewer ultra-processed products [56,57]. In addition, shorter life expectancies may reduce postmenopausal exposure to risk, while differing cultural meanings of illness can lead diseases such as cancer to be either undiscussed or treated with traditional medicine [57,58]. Some associations appear paradoxical when considered from an individual-level biological perspective (e.g., milk consumption or red meat intake). These are more likely to reflect broader socioeconomic, cultural, or systemic factors linked to breast cancer incidence at a population level, rather than direct causal relationships. Such nuances demand caution in interpretation: low incidence should not be viewed solely as a data deficit but may also reflect alternative lifeways in which breast cancer risk is shaped by social configurations that diverge from conventional biomedical assumptions.

The role of educational attainment and health expenditure highlights the complex nature of breast cancer incidence. While both were positively associated with incidence, they likely act as proxies for access to screening and diagnosis, rather than as direct risk factors. Higher education may improve health literacy and preventive behaviors, but it can also correlate with mediating factors such as alcohol use, delayed childbirth, or hormone therapy, particularly in high-income settings. Likewise, greater health expenditure may indicate stronger healthcare infrastructure, which improves detection but may not reflect higher biological risk [59,60]. From an anthropological perspective, the visibility of breast cancer is shaped by social narratives, stigma, and cultural views on femininity and illness [61]. In high-resource settings, awareness is enhanced by advocacy and technology, whereas in low-resource contexts, breast cancer may remain invisible or stigmatized, delaying care-seeking behaviors [62]. Additionally, variables like height—often a marker of early-life nutrition and socioeconomic position—may point to deeper structural inequalities and unmeasured risk factors not captured by education alone. Thus, while these variables are not modifiable, they serve as crucial indicators to guide prevention strategies, risk communication, and equitable screening policies, especially in underserved populations [63,64,65].

Notably, discontinued breastfeeding stood out as the most powerful individual predictor of breast cancer incidence. While its protective role is well documented through hormonal and cellular mechanisms, its significance also lies in its cultural embeddedness. In many societies, breastfeeding is an act situated within kinship expectations, religious values, and economic necessity, not merely a biomedical recommendation. Its decline in high-incidence settings reflects deeper shifts in maternal identity, labor markets, and gender dynamics, changes that influence not just lactation practices but entire life trajectories that intersect with health risk [66].

The inclusion of cocaine use as a predictor further illustrates the need for multidimensional interpretation. While the biological link to breast cancer remains uncertain, its presence in the model likely reflects broader patterns of urbanization, socioeconomic stress, and risk behavior. This highlights the value of including contextual markers that capture underlying social transformations, rather than assuming direct causality. In this sense, drug use may function as a proxy for complex developmental dynamics occurring in certain countries or subpopulations [67,68].

Despite its strengths, the study faces limitations. In first one, giving that this is an ecological analysis at the country level, associations observed here may not reflect individual-level risk “a limitation that requires caution when interpreting these findings. Secondly, the data availability and quality vary significantly across countries, especially in low-income settings, affecting the accuracy of the incidence estimates. While some associations are consistent with literature, others”, such as those involving sanitation “require cautious interpretation (not be misinterpreted as protective effects but rather as context-specific dynamics of society i.e., underdiagnosis and underreporting in low-resource settings)”. A review of related literature shows few direct links between sanitation and breast cancer [66], with some studies instead focusing on associations with endometrial or gastrointestinal cancers [69,70]. This highlights the need to focus on underserved populations in order to strengthen public health measures that improve access to diagnosis, prevention, and culturally appropriate care.

One important limitation is that this study is that part of the data corresponds to the period of the COVID-19 pandemic (2019–2021), during which oncological screening, diagnosis, and treatment schedules were disrupted worldwide. This may have led to underestimation of breast cancer incidence in some regions and could partially influence the associations observed [71]. It would be valuable to conduct future analyses comparing pre-pandemic and post-pandemic periods to determine common factors, identify new emerging factors, and recognize those that have remained stable, which will be essential to address in public health strategies.

The results of this study carry important implications for both research and policy. First, future epidemiological research should move toward more context-sensitive models that integrate not only biomedical data but also cultural, reproductive, and dietary variables specific to each region. Incorporating life history, traditional practices, and local food systems may improve the explanatory power of cancer models and help distinguish genuine protective factors from diagnostic artifacts. From a public health perspective, the findings highlight the need to strengthen breastfeeding promotion programs, regulate the marketing and availability of ultra-processed foods, and expand early detection strategies in underserved areas. However, interventions must be culturally adapted, avoiding one-size-fits-all models and recognizing the diversity of health behaviors across populations. Policies should also address structural inequalities by investing in primary healthcare infrastructure, improving sanitation, and ensuring access to culturally appropriate health education. Ground prevention strategies in both science and cultural understanding can help reduce disparities and promote more equitable global breast cancer control.

## 5. Conclusions

This study developed a reliable and multifactorial model that explains approximately 73% of the global variance in breast cancer incidence, revealing key associations with behavioral, structural, and cultural variables. Positive predictors such as discontinued breastfeeding, processed food consumption, and out-of-pocket health expenditure suggest both lifestyle transitions and greater diagnostic capacity in higher-resource settings. Conversely, negative associations with variables like unsanitary conditions and lack of handwashing facilities likely reflect a combination of underdiagnosis and distinct sociocultural contexts—such as traditional reproductive behaviors, low-calorie diets, and shorter life expectancy.

These findings confirm that breast cancer is embedded in broader social, economic, and cultural systems. Concrete implications for policy include the strengthening of breastfeeding promotion programs, the regulation and monitoring of ultra-processed food consumption, and the expansion of equitable access to preventive healthcare services, especially in underserved populations. By linking these strategies to global health policy framework (WHO’s Global Action Plan for the Prevention and Control of Noncommunicable Diseases), this study underscores the need for context-sensitive interventions that address both modifiable risk factors and structural inequalities. Furthermore, while the present analysis provides a global overview, future studies should incorporate more granular, country-specific data to identify local modifiable factors and guide prevention strategies tailored to regional healthcare systems, cultural practices, and socioeconomic conditions. This approach will strengthen the relevance of the model for policymakers and support targeted interventions while advancing global cancer control.

## Figures and Tables

**Table 1:** Description and Sources of Sociodemographic, Nutritional, and Health-Related Variables Included in the Study Across 183 Countries (2017–2020).

Type of Variable	Variable	Description	Data Source
Epidemiology	Breast cancer incidence	Estimated age-standardized incidence rates in 2020, breast cancer, females, all ages	Global Cancer Observatory (GCO), hosted by the International Agency for Research on Cancer (IARC), World Health Organization (WHO) [3]
Social and Health	Expected years of schooling	The number of years a child of school entrance age can expect to receive if the current age specific enrolment rates persist throughout the child’s years of schooling. 2021	United Nations Development Programme (UNDP)—Human Development Report, processed by Our World in Data [13]
	Out-of-pocket expenditure per capita on healthcare	Estimates the average health expenditure through out-of-pocket payments per capita, indicates how much every person pays out of pocket on average in USD PPP at the point of use. High out of pocket payments are associated with catastrophic and impoverishing household spending. 2019	World Health Organization (WHO) via World Bank, processed by Our World in Data [14]
	Drug use	The drug use risk factor includes the risk of suicide in prevalent cases of opioid, amphetamine, and cocaine use disorders, as well as the cumulative incidence of bloodborne infections due to current and past injection drug use.	Institute for Health Metrics and Evaluation (IHME) and Global Burden of Disease Study (GBD) 2019 [15]
	Unsafe water source	Women of all ages exposed to unsafe water at its primary source, 2019. (Rate exposure per 100)	
	Unsafe sanitation	Females exposed to unsafe sanitation based on the primary toilet type used, 2019. (Rate of exposure per 100)	
	No access to handwashing facility	Female exposure to no access to handwashing facility with available soap and water, 2019. (Rate per 100)	
	Prevalence of Cocaine use	Annual Prevalence (percentage) of the use of cocaine, by region and globally. Cocaine includes cocaine salt, “crack” cocaine and other types such as coca paste, cocaine base, basuco, paco and merla. Data period could include years 2015–2021	United Nations Office on Drugs and Crime (UNODC) [16]
Nutritional	Alcohol consumption per person	Alcohol consumption per person, 2018. Consumption of alcohol is measured in liters of pure alcohol per person aged 15 or older, per year.	Our World in Data, using data from World Health Organization (WHO) [17]
	BMI (kg/m^2^)	Mean BMI (kg/m^2^) (age-standardized estimate) Female 2019	IHME, GBD 2019, and WHO [18]
	High fasting plasma glucose	Female exposure to high fasting plasma glucose among all age groups in 2019, values represented by the rate exposure per 100 individuals.	IHME (The Institute for Health Metrics and Evaluation) with Global Burden of Disease (GBD) study 2019 [15]
	High LDL cholesterol	Female exposure to high LDL cholesterol levels across all age groups in 2019 is presented as the rate of exposure per 100 individuals.	
	High systolic blood pressure	Female exposure to high systolic blood pressure across all age groups in 2019, values represented by the rate of exposure per 100 individuals.	
	Low bone mineral density	Female exposure to low bone mineral density among all age groups in 2019, presented as rate of exposure per 100 individuals.	
	Kidney dysfunction	Female exposure to kidney dysfunction across all age groups in 2019 presented as the rate of exposure per 100 individuals. is defined as estimated glomerular filtration rate (eGFR) <60 mL/min/1.73 m^2^ or albumin to creatinine ratio (ACR) ≥30 mg/g. The theoretical minimum risk exposure level value is ACR <30 mg/g and eGFR ≥60 mL/min/1.73 m^2^.	Institute for Health Metrics and Evaluation (IHME) with Global Burden of Disease (GBD) study 2019 [15]
	Iron deficiency exposure	Female exposure to iron deficiency among all age groups in 2019 presented as the rate of exposure per 100 individuals. Defined as iron deficiency exposure was operationalized as the modeled population mean hemoglobin for a given location, year, age, and sex.	
	Zinc deficiency	Female exposure to zinc deficiency across all age groups in 2019 presented as the rate of exposure per 100 individuals.	
	Vitamin A deficiency	Female exposure to vitamin A deficiency across all age groups in 2019 presented as the rate of exposure per 100 individuals.	
	Fruit consumption per capita	Fruit consumption per capita, 2020. Average fruit consumption per person, measured in kilograms per year (kg/person/year).	Our World in Data, using data from World Health Organization (WHO) [19,20]
	Vegetable consumption per capita	Vegetable consumption per capita, 2020. Average per capita vegetable consumption, measured in kilograms per person per year (kg/person/year).	
	Cereals and grains kilocalories per day per capita	Average daily kilocalories consumption by cereals and grains (2020): “This data represents the daily per capita supply of calories categorized by food group, specifically cereals and grains, for all age groups in the year 2020.	Food and Agriculture Organization of the United Nations (FAO) and Our World in Data [21]
	Sugar consumption kilocalories per day	This data represents the daily per capita supply of calories from sugar, measured in kilocalories, for the year 2020.	Our World in Data, using data from World Health Organization (WHO) [22,23]
	Dairy and eggs kilocalories per day per capita	Represents the daily per capita supply of calories categorized by food group, specifically dairy (milk) and eggs, for all age groups in the year 2020.	
	Oils and fat kilocalories per day per capita	This data represents the average daily per capita supply of dietary fat, measured in grams per person per day, for the year 2020.	
	Diet high in red meat	Female exposure to a diet high in red meat across all age groups in 2019 is presented as the rate of exposure per 100 individuals. Defined as intake above an average of 0 g per day (95% UI 0–200) of unprocessed red meat. Unprocessed red meat includes pork and bovine meats such as beef, lamb, and goat, but excludes all processed meats, poultry, fish, and eggs.	
	Diet high in processed meat	Female exposure to a diet high in processed meat across all age groups in 2019 is presented as the rate of Summary Exposure Value (SEV) per 100 individuals. Diet high in processed meat is defined as any intake (in grams per day) of meat preserved by smoking, curing, salting, or addition of chemical preservatives.	Institute for Health Metrics and Evaluation (IHME) and Global Burden of Disease Study (GBD) [19,23]
	Seafood omega-3 fatty acids consumption	Defined as average daily consumption (in milligrams per day) of less than 470–660 milligrams of eicosapentaenoic acid (EPA) and docosahexaenoic acid (DHA) from seafood sources.	
	Polyunsaturated fatty acids consumption	Defined as average daily consumption (in % daily energy) of less than 9–10% total energy intake from omega-6, specifically linoleic acid, γ-linolenic acid, eicosadienoic acid, dihomo-γ-linolenic acid, and arachidonic acid.	
	Diet high in trans fatty acids	Female exposure to a diet high in trans fatty acids across all age groups in 2019 is presented as the rate of exposure per 100 individuals. Defined as intake greater than 0–1·1% daily energy of trans fat from all sources, mainly partially hydrogenated vegetable oils and ruminant products.	
	Diet high in sugar-sweetened beverages	Female exposure to a diet high in sugar-sweetened beverages across all age groups in 2019 is presented as the rate of exposure per 100 individuals. Defined as any intake (in grams per day) of beverages with ≥50 kcal per 226·8 g serving, including carbonated beverages, sodas, energy drinks, and fruit drinks, but excluding 100% fruit and vegetable juices.	
	Milk consumption	Defined as average daily consumption in grams per day from all dairy milk sources, including non-fat, low-fat, and full-fat, and excluding soy milk and other plant derivatives. The optimal intake for females is defined as 500–610 g per day	
	Calcium intake	Calcium intake is defined as average daily consumption of dietary calcium in grams per day from all sources, including milk, yogurt, and cheese.	
	Non-exclusive breastfeeding	Female exposure to non-exclusive breastfeeding in 2019 is presented as rate per 100 individuals.	
	Discontinued breastfeeding	Female exposure to discontinued breastfeeding in 2019 is presented as the rate of exposure per 100 individuals. (refers to the process in which a mother stops breastfeeding her child or less than 6 months of breastfeeding).	

The data correspond to the period from 2019 to 2023 and were obtained from international databases, including FAO and WHO. These estimates cover 183 countries and provide a global perspective on the analyzed variables. Values are average and may not capture specific variations within each country.

**Table 2:** Comparison of Various Mathematical Models Identified in the Multiple Regression Analysis.

Model	R	R^2^	Adjusted R^2^	Standard Error of Estimate	Change in R Square	Contribution Per Variable (Coefficients)	
1	0.754	0.538	0.565	15.075	0.568	Constant	15.407
						Discontinued breastfeeding	2.522
2	0.796	0.634	0.629	13.931	0.065	Constant	14.407
						Discontinued breastfeeding	2.226
						Prevalence of Cocaine use	7.811
3	0.826	0.682	0.675	13.029	0.048	Constant	32.170
						Discontinued breastfeeding	1.488
						Prevalence of Cocaine use	7.330
						Unsafe sanitation	−0.251
4	0.843	0.710	0.702	12.477	0.028	Constant	31.338
						Discontinued breastfeeding	1.013
						Prevalence of Cocaine use	7.179
						Unsafe sanitation	−0.202
						Out-of-pocket expenditure per capita on healthcare	0.017
5	0.850	0.722	0.713	12.257	0.012	Constant	28.833
						Discontinued breastfeeding	1.036
						Prevalence of Cocaine use	7.795
						Unsafe sanitation	−0.337
						Out-of-pocket expenditure per capita on healthcare	0.018
						No access to handwashing facility	0.160
6	0.855	0.731	0.721	12.085	0.010	Constant	29.396
						Discontinued breastfeeding	0.893
						Prevalence of Cocaine use	7.273
						Unsafe sanitation	−0.369
						Out-of-pocket expenditure per capita on healthcare	0.013
						No access to handwashing facility	0.161
						Diet high in processed meat	0.123

The predictors identified in the multiple regression analysis, considering the significance level of *p* < 0.05. R = multiple correlation coefficient; R^2^ = coefficient of determination; Adjusted R^2^ = R^2^ adjusted for the number of predictors and sample size.

## Data Availability

The original contributions presented in the study are included in the article; further inquiries can be directed to the corresponding author.
